# Impact of sleep duration on bone mineral density and osteoporosis risk: A systematic review and meta-analysis

**DOI:** 10.12669/pjms.42.6.16122

**Published:** 2026-06

**Authors:** Weihong Xiong, Weifeng Li

**Affiliations:** 1 Weihong Xiong, Department of Orthopedics, Changxing County People’s Hospital, Huzhou City, Zhejiang Province 313100, P.R. China; 2 Weifeng Li, Department of Orthopedics, Changxing County People’s Hospital, Huzhou City, Zhejiang Province 313100, P.R. China

**Keywords:** Osteoporosis, Sleep, Bone Mineral Density, Association, Meta-analysis

## Abstract

**Background and Objective:**

Poor sleep has been linked to numerous adverse health outcomes. However, the impact of sleep quality on bone health remains unclear. This systematic review and meta-analysis aimed to evaluate the link between the duration of sleep and the risk of osteoporosis and low bone mineral density (BMD).

**Methodology:**

A comprehensive literature search was performed in PubMed, EMBASE, the Cochrane Library, and Scopus from inception till June 30, 2025 to identify studies examining the relationship between sleep duration and bone health outcomes. The primary effect measures were odds ratios (ORs) with 95% confidence intervals (CIs) for osteoporosis and low BMD. Pooled estimates were calculated using random-effects models, and both qualitative and quantitative syntheses were conducted.

**Results:**

Thirty eligible studies were included. Sleeping ≤6 hours was associated with high risk of osteoporosis (OR 1.58; 95% CI, 1.29–1.94; I^2^=35%) and low BMD (OR 1.49; 95% CI, 1.09–2.03; I^2^=81%). Sleep durations of 6–8 hours were not significantly associated with osteoporosis risk (OR 1.06; 95% CI, 0.94–1.19; I^2^=76%). In contrast, sleeping 8–9 hours was associated with a moderate increase in risk (OR 1.25; 95% CI, 1.11–1.40; I^2^=86%), while sleeping ≥9 hours showed a non-significant trend toward increased risk (OR 1.38; 95% CI, 0.95–2.01; I^2^=69%). Subgroup analyses of cohort studies demonstrated consistent associations with lower heterogeneity.

**Conclusions:**

Both short and long sleep durations are associated with an increased risk of osteoporosis and low bone mineral density, whereas a sleep duration of 6–8 hours appears to be optimal for bone health. Sleep duration emerges as a potentially modifiable target for osteoporosis prevention and warrants further mechanistic and interventional research.

***Prospero Registration Number:*** CRD420251134319.

## INTRODUCTION

Osteoporosis is a common age-related bone disorder characterized by reduced bone mineral density (BMD), abnormalities in bone microarchitecture, and an increased risk of fractures.^1^ The International Osteoporosis Foundation reports that approximately one in three women and one in five men over 50 years of age experience osteoporotic fractures.^2^ In addition to associated substantial morbidity and mortality, osteoporotic fractures are associated with a significant economic burden.^3^,^4^ Therefore, identifying risk factors for osteoporotic fractures and timely preventive measures are crucial to reduce associated complications.^5^

Sleep disturbances have long been linked to adverse health outcomes, such as cognitive decline, cardiovascular diseases, and metabolic disorders.^6^-^8^ The mechanisms of these associations include hormonal imbalance and inflammatory processes.^8^ While several studies have shown the link between sleep duration and bone health,^9^,^10^ the reported association has been inconsistent due to heterogeneity across studies (e.g., study design, age groups, adjustment for confounders). Some studies reported that longer sleep duration correlates with increased risk of low BMD/osteoporosis,^11^,^12^ suggesting that prolonged sleep may negatively affect bone health. A meta-analysis that included data on middle-aged and elderly patients found a higher risk of osteoporosis among individuals who reported sleeping eight hours or more per day.^13^ However, several studies have reported that short sleep duration has been associated with an increased risk of osteoporosis and lower BMD, suggesting potential detrimental effects of insufficient sleep.^14^

The meta-analysis by Wang et al.^15^ examined the association between sleep duration and osteoporosis but included data from only four studies, thereby limiting the robustness of findings. Additionally, that analysis did not address the association between low BMD and sleep duration, which is an osteoporosis precursor and an important clinical outcome in its own right. We designed this study to address the limitations of the earlier meta-analysis and take advantage of the increasing evidence in this area, to obtain an updated comprehensive evaluation of the available evidence. Recognizing sleep duration as a potentially modifiable risk factor for osteoporosis underscores the relevance of sleep hygiene interventions as adjunctive strategies in the prevention and long-term management of impaired bone health.

## METHODOLOGY

**Fig.1:**
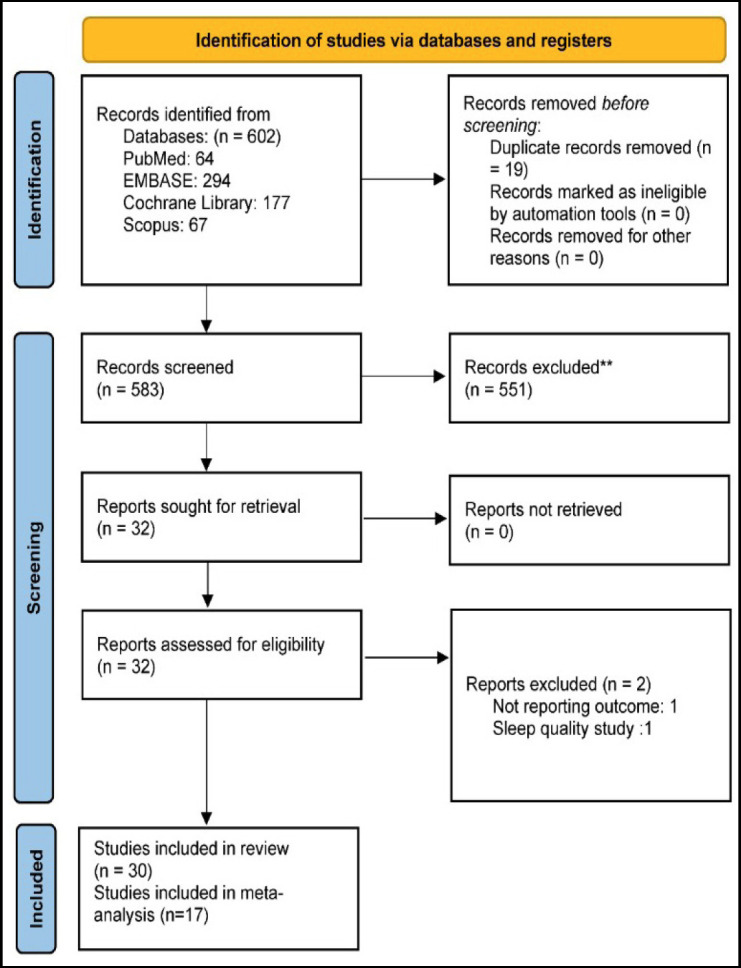
Study Selection Flow Chart.

The systematic review and meta-analysis was carried out following the Preferred Reporting Items for Systematic Reviews and Meta-Analysis (PRISMA) guidelines,^16^ Fig.1. The protocol for the study was registered in PROSPERO (registration number, CRD420251134319).

### Research question:

How does sleep duration affect the risk of osteoporosis and BMD of adults?

### Search strategy:

A comprehensive literature search was carried out by two independent reviewers (W.X and W.L) electronically across multiple digital databases (PubMed, Embase, Cochrane Library, and Scopus) from inception till June 30, 2025 and manually across relevant journals like *Journal of Bone and Mineral Research*; *Osteoporosis International*; *Sleep*; *Journal of Clinical Endocrinology & Metabolism* and *Scientific Reports*. The keywords and MeSH terms related to bone health and sleep duration were combined, such as “sleep duration,” “osteoporosis”, and “bone mineral density,”. The detailed search strategy employed is as follows: ((“Sleep”[Mesh] OR “sleep duration” OR “sleep time” OR “sleep patterns” OR “short sleep” OR “long sleep”) AND (“Bone Density”[Mesh] OR “bone mineral density” OR “BMD”) AND (“Osteoporosis”[Mesh] OR “osteoporosis” OR “bone health”)). The search was then adapted to each database and the records were retrieved (Supplementary Table I). The search was limited to studies published in English until June 2025. Additionally, the reference lists were searched to identify any further eligible studies.

**Supplementary Table-I:** Search strategy in digital databases

*Database*	*Search String*	*Records*
PubMed	((“Sleep”[Mesh] OR “sleep duration” OR “sleep time” OR “sleep patterns” OR “short sleep” OR “long sleep”) AND (“Bone Density”[Mesh] OR “bone mineral density” OR “BMD”) AND (“Osteoporosis”[Mesh] OR “osteoporosis” OR “bone health”))	64
EMBASE	(‘sleep’/exp OR ‘sleep duration’ OR ‘sleep time’ OR ‘sleep patterns’ OR ‘short sleep’ OR ‘long sleep’) AND (‘bone mineral density’/exp OR ‘bone mineral density’ OR ‘BMD’) AND (‘osteoporosis’/exp OR ‘osteoporosis’ OR ‘bone health’)	294
Cochrane Library	((MeSH descriptor: [Sleep] explode all trees OR “sleep duration” OR “sleep time” OR “sleep patterns” OR “short sleep” OR “long sleep”) AND (MeSH descriptor: [Bone Density] explode all trees OR “bone mineral density” OR “BMD”) AND (MeSH descriptor: [Osteoporosis] explode all trees OR “osteoporosis” OR “bone health”))	177
Scopus	(TITLE-ABS-KEY (“sleep duration”) OR TITLE-ABS-KEY (“sleep time” ) OR TITLE-ABS-KEY (“sleep patterns” ) OR TITLE-ABS-KEY (“short sleep” ) OR TITLE-ABS-KEY (“long sleep” ) ) AND (TITLE-ABS-KEY (“bone mineral density” ) OR TITLE-ABS-KEY (“BMD” ) ) AND (TITLE-ABS-KEY (“osteoporosis” ) OR TITLE-ABS-KEY (“bone health” ) )	67

The records identified were imported to a citation manager (Endnote V17.0, Clarivate Analytics, USA) and duplicates were removed.

### Study selection:

Two independent reviewers conducted the study selection on the basis of the initial screening of abstracts and titles. The potentially eligible articles were subjected to full-text assessment and pre-defined inclusion and exclusion criteria were applied.

### Inclusion criteria:

- Observational (cross-sectional, case-control, and cohort) and interventional studies.
- Studies reporting on the association between osteoporosis and/or BMD with sleep duration.
- Studies with enough data to calculate relative risks (RRs), odds ratios (ORs), or hazard ratios (HRs) with 95% confidence intervals (CIs).

### Exclusion criteria:

- Studies on animals
- Reviews, case reports, editorials, and conference abstracts.
- Participants under any medications related to osteoporosis.
- Studies without sufficient data or not reporting BMD or osteoporosis outcomes.

### Data extraction:

Two reviewers used a standardized extraction form to collect the following information from the studies: Author(s) names, country of study, year of publication, study design and sample size, participant characteristics (age, sex), follow-up lengths, measures of sleep duration categories, BMD and osteoporosis assessment tools, outcomes related to BMD and/or osteoporosis, adjusted and unadjusted risk estimates (ORs with 95% Cis), and adjusted confounders. Any reviewers’ disagreements were resolved via discussions.

### Quality of included studies:

Two reviewers independently assessed the quality of each study using the Newcastle-Ottawa Scale (NOS) for cohort studies. The scale evaluates the selection of study groups, the ascertainment of either the exposure or outcome of interest, and the comparability of groups. Stars were awarded if the study met the criteria for each domain item, with a maximum of nine stars for cohort studies. Higher scores indicated higher methodological quality with a lower risk of bias. A score more than six was generally considered to be of good quality.

### Data Synthesis:

The extracted data were synthesized descriptively, summarizing the main findings of the included studies. The primary outcome was the association between the risk of low BMD or osteoporosis and the sleep duration. The studies were grouped according to the categories of sleep duration and analyzed them accordingly. Revman 5.4 v (Cochrane Collaboration, UK) was used to perform the meta-analyses and calculated pooled risk estimates (OR) and 95% CIs and used a random-effects model due to the expected study heterogeneity. The *I^2^* statistic values above 50% was considered as indicating substantial heterogeneity. The forest plots were generated to visually represent the results and conducted sub-group analyses to explore potential sources of heterogeneity on the basis of sleep duration, study design, and gender. The publication bias was assessed by visually inspecting funnel plots and considering asymmetries as indicative of potential publication bias.

## RESULTS

The digital searches yielded 602 items. No articles were found in the manual search. The screened titles and abstracts of 583 records (after removing duplicates) was carried out. The two investigators assessed the full texts of 32 reports for eligibility. Two reports were excluded because one did not report the relevant outcome and one focused on sleep quality rather than sleep duration. Finally, 30 studies were included in the systematic review, of which 17 were included in the meta-analysis.

A total of 30 studies^3^,^11^,^12^,^14^,^17^–^42^ conducted sometime between 2007 and 2024 across various countries, using different study designs (20 cross-sectional, 8 cohort, and 2 prospective studies) were included. The total sample size was 152,058, with approximately 94,095 women and 42,662 men. Participants’ ages ranged from 34 to 83 years, and follow-up periods, reported in Nine studies, ranged from 5 to 10.7 years. Geographically, the studies span the USA (nine studies), China (eight studies), and other places, including Mexico, Germany, Taiwan, Japan, UK, Iceland, Netherlands, France, Korea, India, and Australia. Cross-sectional studies dominated the landscape, particularly in the USA and China, reflecting the significant focus on health research in these regions. The studies showed different sample sizes, from 59 to 31,769 participants, and a high representation of female participants at 61.88% overall.

Low sleep duration was often defined as less than six or seven hours per night across all studies, with sleep categories mostly corresponding to less than six hours, six to eight hours, eight to nine hours, and above nine hours. Both long and short sleep durations were consistently associated with decreased BMD and an increased risk of osteoporosis. BMD was primarily measured using DXA scans. Low BMD references typically used T-scores below -1 or -2.5 at different skeletal sites. Outcome measures included BMD at various sites, fracture risks, and markers of bone turnover. Studies adjusted for a wide range of confounding factors, such as age, BMI, sex, smoking status, physical activity, alcohol consumption, comorbidities, dietary intake, and menopausal status. The NOS scores ranged between seven and nine indicating good study quality (Supplementary Table-II).

**Supplementary Table-II:** Demographic characteristics for included studies.

Author Year Location	Study Design	Study Date (year)	Sample size	Male Patients	Female Patients	Age (Mean/ Median) in years	Follow- up	Low Sleep Reference	BMD assessment tool	Low BMD reference	Osteoporosis Assessment tool	Outcome measures	Confounding factors adjusted	NOS score
Mene-ses-León et al. 17 2024 Mexico	Prospective longitudinal cohort study	2004–2012	1,337	341	996	46 (37–55)	2 years	Sleeping < 7 h/day	DXA scan	T-score below −1 at the lumbar spine and total hip	NR	Low-BMD at different sites	BMI, smoking status (never, former smoker), calcium supplements, calcium intake, Type 2 diabetes, hormone replacement therapy, sleep duration and napping	8
Petrov et al. 18 2024 USA	Cross-sectional study	NR	59	29	30	18-25	1 year	NR	DXA Scan	NR	NR	Bone turnover and BMD	NR	8
Rassow et al.^19^ 2024 Germany	Population-based cohort study	2008–2012	1037	568	469	53.0 (43.0–63.0)	NR	NR	NR	NR	Self- reported osteoporosis	Primary outcome: -two bone turnover marker concentrations and quantitative ultrasound-based stiffness index; secondary outcome: self-reported osteoporosis	Age, sex, BMI, alcohol consumption, smoking, and physical inactivity	9
Chen et al. 20 2024 Taiwan	Cross-sectional study	2008–2018	13,330	4681	8649	55.49 ± 10.22	NR	NR	DXA scan	NR	BMD T-score	Sleep duration; physical activity	Age, gender, weight, systolic blood pressure, HbA1C, fasting and post-prandial glucose, total cholesterol, triglycerides, creatinine, uric acid, and diabetes.	9
Ma et al. 21 2023 China	Retrospective cross- sectional study	NR	148	NA	148	66.26 ± 8.83	NR	Sleeping < 7 h/day	DXA scan	T-score <-1.0 SD, but >-2.5 SD	BMD T-score	The association between sleep duration and osteoporosis	NR	8
Zhao et al. 22 2023 China	Large, multi- centred Cohort study	Baseline survey 2016–2017 and follow-up July 2018 to September 2021	8033	3001	5032	55.8 ± 10.8	3 years	Sleeping < 7 h/day	CM-300, DXA scan	NR	percentage of young adult mean	BMD	Age, body mass index, physical activity, causative diseases, smoking status, and alcohol intake for men and women and further adjusted for menopausal status for women	7
Yamaura et al.^23^ 2023 Japan	Cross-sectional study	May 2020 to July 2022	1717	896	821	M= 54 (46, 64), F= 55 (46, 64)	NR	NR	Ultrasonic bone density apparatus	NR	Osteoporosis self-assessment tool for Asians	Sleep duration; bone quality	Age, sex, marital status, education levels, average monthly income, smoking status, drinking status, nap duration, sleep efficiency scores, physical activity, BMI, and menopausal status (only for women)	7
Tang et al. 12 2022 USA	Cross-sectional study	Examination Survey: 2007–2014	4399	2066	2333	55.00 (46.00–65.00)	NR	NR	DXA scan	NR	BMD T-score	Altered sleep duration and quality on bone mineral density, trabecular microarchitecture, and biochemical markers of bone turnover	NR	8
Zeng et al.^24^ 2022 China	Cross-sectional study	February 2020 to September 2021	169	NR	148	71.91 ± 10.26	NR	NR	DXA scan	NR	NR	Sleep duration and BMD	Age, sex/menopause status, race, income level, BMI, smoking status, alcohol drinking status, physical activity level, fractures history, glucocorticoid use, family history of osteoporosis, calcium intake, and serum 25-hydroxyvitamin D	8
Cherian et al. 25 2022 India	Prospective study	March 2019 to August 2021	190	NA	190	58.2 ± 6.9	2 years	NR	DXA scan	Lumbar spine T- Score below −3.27 (1.76)	BMD T-score	BMD T score assessed at the lumbar spine (L1–L4)	Age, sex, and BMI	8
Tang et al. 26 2021 USA	Cross-sectional study	Examination Survey: 2017–2018	1865	989	876	64.67 ± 8.98	NR	NR	DXA scan	T score < − 1.0 and > − 2.5	BMD T-score	BMD and sleep duration/quality	Age, gender, energy intake, chronic kidney disease status, and body weight	8
Pan et al. 27 2021 Australia	Longitudinal and population-based prospective cohort study	NR	1000	480	520	62.9	2.6–5.1 and 10.7 years	NR	DXA scan	NR	NR	BMD, Falls risk score [physiological profile assessment], fracture and covariate measurements	Age, sex, body mass index, physical activity, smoking history, presence of any comorbidities and multisite pain	8
Lee et al. 11 2021 USA	Cross- sectional study	Examination Survey: 2005–2010	12793	6623	6170	46.25 (45.57– 46.94)	NR	Sleeping < 6 h/day	DXA scan	NR	NR	Bone mineral density; Bone turnover markers	Age, race, clinic site, BMI, alcohol use, GFR, number of hours of sleep needed to feel rested, daily naps, overall health, and use of calcium, androgens, anti-androgens	8
Swanson et al. 28 2021 USA	Cross-sectional study	NR	1055	1055	NA	77	NR	NR	DXA scan	NR	BMD Score	Sleep patterns; BMD	Age, sex, race, education level, income to poverty ratio, BMI, smoking status, alcohol consumption status, hypertension, diabetes, physical activity, RA, cancer or malignancy, use of prednisone or cortisone	8
Ochs-Balcom et al.^14^ 2020 USA	Cross-sectional study	NR	11,084	NA	11,084	63.3 ± 7.4	NR	NR	DXA scan,	NR	NR	Bone related outcome	Age, BMI, social class, smoker status (never, ex-smoker or current smoker), alcohol consumption (units per week), physical activity, dietary calcium intake, number of comorbidities and, in women, years since menopause and hormone replacement therapy (HRT) use	8
Wu et al. 3 2020 China	Cross-sectional study	January 2013 to August 2013	3659	NA	3659	60 ± 8	NR	Sleeping < 6 h/day	DXA scan	T-score between −2.5 and −1	T- score ≤ −2.5	Sleep duration and quality; bone mineral density (BMD)	Age, DXA machine, race, menopausal symptoms, education, smoking, physical activity, body mass index, alcohol use, physical function, and sleep medication use.	8
Bevilacqua et al.^29^ 2020 UK	Cross- sectional study	2011–2012	323	169	154	M= 75.4 ± 2.5; F= 75.7 ± 2.6	NR	NR	NR	NR	Osteoporosis self- assessment tool for Asians	NR	Age at menopause, BMI, education level, nation, currently smoking, currently drinking, currently taking oestrogen, physical activity, hypertension, diabetes mellitus, history of hyperthyroidism, Lipid Profile	8
Swanson et al.^30^ 2019 USA	Prospective cohort study	January 2002 to April 2004	874	NA	874	**Short sleep=** 83.0 ± 3.3; **recommended sleep=** 83.5 ± 3.4	NR	Sleeping < 6 h/day	DXA scan	NR	NR	Sleep duration; bone mineral density (BMD)	Age, race, clinical site, BMI, Calcium or vitamin D use, depression, walking speed, HTN, COPD, daily naps, hours of sleep needed to feel rested, oestrogen use	8
Marques et al.^31^ 2017 Iceland	Single-center prospective population study	2002 – 2006	5764	2438	3326	77	NR	NR	DXA scan	NR	T-score ≤−2.5	Sleep duration, sleep timing and sleep quality and osteopenia and sarcopenia	Age, whole body fat mass, ethnicity, education, alcohol intake, physical activity, vitamin D levels and season, usage of systemic corticosteroids and bisphosphonates	8
Lucassen et al. 32 2017 Netherlands	Population-based prospective cohort study	September 2008 to September 2012	915	403	512	59 ± 4	NR	Sleeping < 6 h/day	Volumetric Quantitative Computed Tomography	NR	NR	Bone and muscle related outcome	Age, BMI, physical activity level, smoking status, health status, mobility disability status, number of medical conditions, cognitive status, hs-CRP, and bone medication (only in the BMD predictor model)	8
Saint Martin et al.^33^ 2016 France	Cross- sectional (prospective) study	2001–2003	500	210	290	65.7 ± 0.8	NR	Sleeping < 6 h/day	DXA scan	NR	T score < − 1.0 and > − 2.5	Sleep duration and quality; bone mineral density (BMD); physical activity on osteopenia and osteoporosis	Gender, BMI, presence or absence of dyslipidaemia and mean daily energy expenditure	9
Cunningham et al. 36 2015 America	Population- based cross- sectional study	Examination Survey: 2005–06 and 2007–08	5288	NR	2654	66.1 ± 10.2	NR	Sleeping < 6 h/day	DXA scan	NR	BMD ≤ −2.5	Femoral neck BMD	Race, sex, BMI, Average sleep duration, Family history of osteoporosis, Arthritis, General health condition, Chronic bronchitis, any sleep disorder, and history of fractures	8
Niu et al. 35 2015 US	Cross- sectional study	Baseline: June 2004 to October 2009	750	207	543	45-75	2 years	Sleeping < 6 h/day	DXA scan	NR	NR	BMD	Age, education level, smoking, physical activity, depressive symptomatology, comorbidity and serum vitamin D concentration	8
Tian et al. 34 2015 China	Cross- sectional study	NR	31769	13,533	16,851	M= 67.60 ± 6.76; F= 61.59 ± 8.73	NR	NR	Calcaneal quantitative ultrasonography (QUS)	NR	BMD ≤−2.5	Sleep duration, sleep timing and risk of osteoporosis	Smoking status, alcohol drinking status, regular physical activity, hypertension, and diabetes mellitus in males and menopause status, and age at menopause in women	8
Wang et al. 37 2015 China	Multicentre, prospective, observational study	NR	6510	NA	6510	58.25 ± 10.32	NR	NR	quantitative ultrasound index in calcaneal quantitative ultrasonography	NR	BMD <−2.5	Bedtime, total sleep duration, nocturnal sleep duration, daytime sleep duration, noontime napping duration and BMD [ T- Score ]	Age at baseline, education level, occupation, cigarette smoking status, regular exercise, HbA1c value, diabetes diagnosed, BMI, calcium tablet/vitamin D supplement, milk intake, oral contraceptive use or oestrogen supplement, history of osteoporosis, history of fractures, histories of hyper/hypothyroidism, levothyroxine treatment	8
Chen et al. 38 2014 China	Cross- sectional study	Jun 2011 to January 2012	8688	3950	4738	54.4 ± 10.0	NR	NR	Calcaneal quantitative ultrasonography	NR	T-score ≤ −2.5	Self-reported sleep duration, daytime nap duration, sleep quality, and calcaneus bone mineral density	Age and BMI. Next, adjustment of potentially confounding variables, including lifestyle characteristics (smoking, alcohol consumption, physical activity), chronic diseases (diabetes, dyslipidaemia, hypertension), diets (consumption of milk and soybean products), snoring, and years since menopause (postmenopausal women only)	8
Kim et al. 39 2014 Korea	Cross- sectional study	Study survey: 2008–2010	2679	1,405	1,274	M= 68 ± 0.2; F= 69.1 ± 0.2	NR	Sleeping < 6 h/day	DXA scan	NR	T- score ≤ −2.5	NR	Age, BMI, lifestyle behaviours such as drinking, smoking, physical activity, and coffee consumption, education, serum vitamin D level, calcium consumption, and comorbidities such as hypertension or diabetes mellitus	8
Kobayashi et al. 40 2012 Japan	Large-scale, retrospective cohort study	January 2008 to December 2008	19321	NR	9276	60.9 ± 7.9	NR	Sleeping < 6 h/day	DXA scan	NR	T- score = −2.5 SD	BMD, osteoporosis, and sleep duration	Comorbidities, lifestyle, and physiologic factors, likely through multiple mechanisms involving weight loading and sleep-associated hormonal changes	7
Fu et al. 41 2011 China	Cross- sectional study	2008–2009	602	NA	602	18–80	NR	NR	DXA scan	NR	NR	Total and regional BMD and sleep duration	Age, menopausal status, weight, height, %BF, lifestyle variables, including drinking, smoking, and occupational physical activity	7
Specker et al. 42 2007 US	Cross- sectional study	NR	1146	494	652	20–66	NR	Sleeping < 6.5 h/night	DXA scan	NR	NR	BMD and sleep deprivation	Age, weight, height, percent body fat (by DXA), calcium and vitamin D intake, grip strength, percent time in moderate plus vigorous activity, population group, and oestrogen status (for women)	8

NR- Not Reported; USA- United States of America; UK- United Kingdom; BMD- Bone Mineral Density; DXA- Dual energy Xray Absorptiometry; BMI – Body Mass Index; NR – Not Reported; T- Score- BMD score; HbA1C- Glycated Haemoglobin; GFR- Glomerular Filtration Rate; RA- Rheumatoid Arthritis; HRT – Hormone Replacement Therapy; ESS- Epworth Sleepiness Scale; HTN – Hypertension; COPD – Chronic Obstructive Pulmonary Disease; CRP -C reactive protein, NOS- Newcastle-Ottawa Scale.

### Meta-analysis:

The data from 17 studies^3^,^11^,^12^,^14^,^17^,^20^,^22^,^24^,^25^,^33^–^38^,^40^,^41^ were included into the quantitative analysis. The extracted Ors were pooled using the Generalized Inverse Variance (GIV) model.

### Osteoporosis risk and sleep duration:

**Fig.2:**
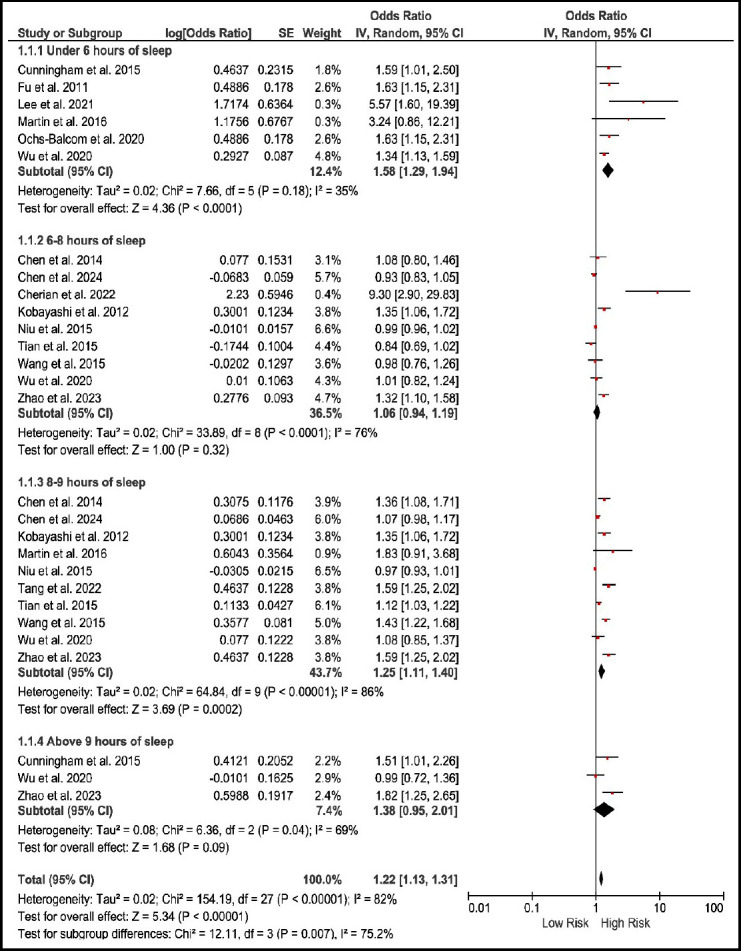
Forest plot showing the pooled odds ratios of the association between sleep duration and the risk of osteoporosis across various studies.

The forest plot assessing the association between the risk of osteoporosis and sleep duration across various studies shows that individuals who sleep 6 hours or less have a significantly increased risk of osteoporosis, with an OR of 1.58 (1.29–1.94); *I^2^*=35%. Sleep durations between six and eight hours do not significantly impact the risk of osteoporosis, with an OR of 1.06 (0.94–1.19); *I^2^*=76%. Conversely, individuals who sleep 8 to nine hours have a moderately increased risk, with an OR of 1.25 (1.11–1.40); I^2^=86%. For individuals who sleep nine hours or more, the risk trend is towards an increase, but it is not statistically significant, with an OR of 1.38 (0.95–2.01); *I^2^*=69%. Overall, when all sleep-duration categories included in the forest plot were pooled, the combined estimate suggested a higher risk of osteoporosis (OR, 1.22; 95% CI, 1.13–1.31). However, this overall estimate should be interpreted cautiously because it combines different sleep-duration categories. The substantial heterogeneity (*I^2^* = 82%) suggests that the impact of sleep duration on osteoporosis risk varies greatly with the number of sleep hours (Fig.2).

### Sleep duration and risk of low BMD:

**Fig.3:**
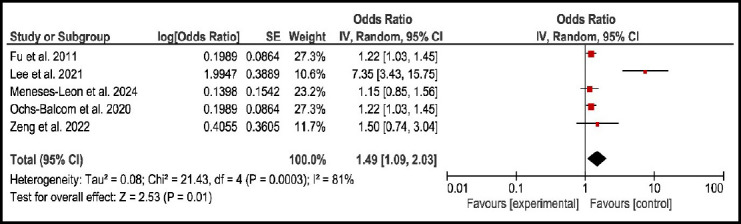
Forest plot showing the pooled odds ratios of the association between sleep duration and the risk of low BMD across various studies.

The pooled analysis across all studies showed a statistically significant 49% increased risk of low BMD for individuals with less than 6 hours of sleep (OR, 1.49; 95% CI, 1.09–2.03), despite substantial heterogeneity (*I^2^* = 81%) among the studies (Fig.3).

### Sub-group analyses:

The subgroup analyses of the association between the risk of osteoporosis and low BMD with sleep duration revealed significant findings. Women sleeping less than six hours had a 43% increased risk of osteoporosis (OR, 1.43; 95% CI, 1.24–1.64), whereas, studies which included both men and women showed a 2.55 higher risk (OR, 2.55; 95% CI, 1.15–5.65). For women with less than six hours of sleep, the risk of low BMD increased by 22% (OR, 1.22; 95% CI, 1.08–1.39). Cohort studies showed significant associations, with a 22% increased risk for those with 6–8 hours of sleep (OR, 1.22; 95% CI, 1.01–1.47; I^2^ = 53%) and a 45% increased risk for those with 8–9 hours of sleep (OR, 1.45; 95% CI, 1.29–1.62; I^2^ = 0%). Cross-sectional studies showed a 58% increased risk of osteoporosis for individuals with less than six hours of sleep (OR, 1.58; 95% CI, 1.29–1.94), but the studies displayed high variability for other durations (Table-I).

**Table-I:** Sub-group analysis based on gender and study design.

Risk	Based on	Sleep	Sub-groups	No. of studies	OR	95% CI	p value	I^2^ value
Risk of Osteoporosis	Gender	<6 hours	Women	3	1.43	1.24–1.64	<0.0001	0%
			Men and Women	3	2.55	1.15–5.65	0.02	51%
		6–8 hours	Women	3	1.37	0.81–2.33	0.24	86%
			Men and Women	6	1.05	0.93–1.18	0.44	75%
		8–9 hours	Women	2	1.26	0.96–1.66	0.1	73%
			Men and Women	8	1.24	1.09–1.40	0.0009	86%
		>9 hours	Women	1	0.99	0.72–1.36	0.95	NA
			Men and Women	2	1.67	1.27,2.2	0.0003	0%
	Study Design	<6 hours	Cohort	0	NA	NA	NA	NA
			Cross-sectional	6	1.58	1.29–1.94	<0.001	35%
		6–8 hours	Cohort	3	1.22	1.01–1.47	0.04	53%
			Cross-sectional	6	0.99	0.87–1.12	0.82	72%
		8–9 hours	Cohort	3	1.45	1.29–1.62	<0.0001	0%
			Cross-sectional	7	1.15	1.03–1.29	0.01	82%
		>9 hours	Cohort	0	NA	NA	NA	NA
			Cross-sectional	3	1.38	0.95–2.01	0.09	69%
Risk for Low BMD	Gender	<6 hours	Women	2	1.22	1.08–1.39	0.001	0%
			Men and Women	3	2.24	0.79–6.41	0.13	90%

## DISCUSSION

Thirty studies^3^,^11^,^12^,^14^,^17^–^42^ were included in the review, encompassing various designs, sample sizes, and demographic characteristics. These studies provided a comprehensive dataset to evaluate the association between bone health and sleep duration.

The present review shows that individuals who sleep six hours or less have a significantly increased risk of osteoporosis. Sleep durations between six to eight hours do not significantly impact the risk of osteoporosis. Conversely, individuals sleeping between eight and nine hours seem to have a moderately increased risk. For individuals who sleep nine hours or more, the risk trend is towards an increase, but without statistical significance. Overall, the pooled estimate across the sleep-duration categories included in the forest plot suggested a higher risk of osteoporosis (OR, 1.22; 95% CI, 1.13–1.31), although this combined estimate should be interpreted cautiously because of substantial heterogeneity and the inclusion of different sleep-duration categories. The substantial heterogeneity (*I^2^*=82%) of the included studies suggests that the impact of sleep duration on the osteoporosis risk varies greatly with the number of sleep hours. For low BMD, the pooled analysis across all studies revealed a statistically significant 49% increased risk for individuals who slept less than six hours of sleep, despite substantial heterogeneity (I^2^=81%) among the studies.

The findings of this study support previous research^,15^ and further show that both short and excessively long sleep durations can negatively impact bone density. The possible mechanisms of this association include hormonal dysregulation, disrupted bone remodeling, increased inflammation, and lifestyle factors.^43^,^44^ Sleep is pivotal for regulating the secretion of several hormones essential for bone health, such as growth hormone (GH) and insulin-like growth factor-1 (IGF-1).^45^ Reduced sleep duration is associated with a negative impact on secretion levels of GH and IGF-1, osteoblast activity, and bone formation.^46^,^47^ Sleep deprivation also elevates cortisol levels, leading to higher osteoclast and lower osteoblast activity, and increased bone resorption.^48^ Additionally, the impact of sleep quality on the parathyroid hormone (PTH) levels and calcium homeostasis further contributes to decreased bone mineral density.^49^

Previous research demonstrated that disrupted sleep patterns affect circadian rhythms, increase bone resorption, and lower the levels of bone formation markers.^50^ Furthermore, reduced sleep lowers melatonin levels, thus impacting osteoblast differentiation and osteoclast activity, and subsequently leading to impaired bone health.^51^,^52^ Chronic sleep deprivation is also associated with high levels of pro-inflammatory cytokines, which stimulate osteoclast activity and inhibit osteoblast function.^53^

Poor sleep has also been linked to changes in patients’ lifestyle, leading to reduced physical activity, poor dietary habits, and increased smoking, and alcohol consumption, all of which have been shown to increase the risk of osteoporosis and low BMD.^54^-^56^

The association between low BMD and sleep duration is evident from the increased risk observed in individuals with shorter sleep durations. Sleep participates in bone remodelling and regeneration processes, and insufficient sleep can impair these functions, leading to decreased bone mass and increased fragility.^9^,^10^

A substantial heterogeneity (*I^2^*=81% for low BMD and *I^2^*=82% for osteoporosis) was found among the included studies, particularly within those of cross-sectional design. Subgroup analyses revealed consistent findings in cohort studies with low heterogeneity, indicating a more reliable association. The variations in findings underscore the importance of considering study design and population characteristics when interpreting results.

### Strengths:

The study’s strengths include the comprehensive inclusion of various study designs and large sample sizes, enhancing the robustness of the findings.

### Limitations:

However, the limitations include highly heterogeneous case-control, cohort, and cross-sectional studies; and the many potential confounding factors that may not have been entirely controlled. Additionally, the comparability of results may be difficult due to variations in sleep assessment methods across studies. Also, potential language bias in selecting English-language articles could miss out few relevant studies. Additionally, there is a heterogeneity in the assessment of BMD and osteoporosis across the included studies. This heterogeneity may be due to the differences in measurement modalities that may differ in sensitivity and specificity (i.e., dual-energy X-ray absorptiometry, quantitative ultrasound, or self-reported diagnoses). DXA remains the gold standard for assessing BMD. In contrast, ultrasound-based methods provide only indirect estimates, and self-reported measures are prone to recall bias and potential misclassification. As a result, there is a real possibility of measurement bias and limited comparability across studies, which may partly explain the observed heterogeneity. The inclusion of both adjusted and unadjusted effect estimates introduces the possibility of residual confounding. Future studies should consistently adjust for key confounders such as age, sex, body mass index, and lifestyle factors.

## CONCLUSION

This study suggests that short sleep duration is associated with increased risks of low BMD and osteoporosis, while longer sleep duration, particularly 8–9 hours, may also be associated with an increased risk of osteoporosis. Adequate sleep is crucial for maintaining bone health. Future studies are needed to clarify the underlying mechanisms of these associations and to develop effective preventive strategies.

### Recommendations:

Further research should focus on conducting well-designed prospective cohort studies and randomized interventional trials to clarify whether the association between sleep duration and bone health is causal. More standardized and objective measurements, such as actigraphy or polysomnography for sleep and DXA for BMD, are needed to reduce variability across studies. Important modifiers, such as age, sex, menopausal status, and comorbid conditions, should be used to clarify how these associations differ across populations. In addition to sleep duration, future studies should focus on the impact of sleep quality on bone health. Improving sleep habits may represent a simple and practical avenue for supporting bone health and potentially reducing osteoporosis risk.

### Authors’ contributions:

**WX:** Literature search, study design and manuscript writing.

**WX** and **WL:** Data collection, data analysis and interpretation.

**WX:** Manuscript revision and validation and is responsible for the integrity of the study.

All authors have read and approved the final manuscript.

## References

[1] Sözen T, Özışık L, Başaran NÇ (2017). An overview and management of osteoporosis. Eur J Rheumatol.

[2] Curtis EM, Van der Velde R, Moon RJ, Van den Bergh JPW, Geusens P, De Vries F (2016). Epidemiology of fractures in the United Kingdom, 1988-2012: Variation with age, sex, geography, ethnicity and socioeconomic status. Bone.

[3] Wu S, Wang P, Guo X, Sun G, Zhou Y, Li Z (2020). The associations between different sleep patterns and osteoporosis based on the Osteoporosis Self-Assessment Tool for Asians. Arch Osteoporos.

[4] Teng GG, Curtis JR, Saag KG (2008). Mortality and osteoporotic fractures: is the link causal, and is it modifiable?. Clin Exp Rheumatol.

[5] General (US) O of the S. Assessing the Risk of Bone Disease and Fracture (2004). In: Bone Health and Osteoporosis: A Report of the Surgeon General. Office of the Surgeon General (US).

[6] Zielinski MR, McKenna JT, McCarley RW (2016). Functions and Mechanisms of Sleep. AIMS Neurosci.

[7] Zheng NS, Annis J, Master H, Han L, Gleichauf K, Ching JH (2024). Sleep patterns and risk of chronic disease as measured by long-term monitoring with commercial wearable devices in the All of Us Research Program. Nat Med.

[8] Nagai M, Hoshide S, Kario K (2010). Sleep Duration as a Risk Factor for Cardiovascular Disease- a Review of the Recent Literature. Curr Cardiol Rev.

[9] Swanson CM, Kohrt WM, Buxton OM, Everson CA, Wright KP, Orwoll ES (2018). The Importance of the Circadian System &Sleep for Bone Health. Metabolism.

[10] Fertelli TK, Tuncay FO (2019). Fatigue in individuals with knee osteoarthritis: Its relationship with sleep quality, pain and depression. Pak J Med Sci.

[11] Lee CL, Tzeng HE, Liu WJ, Tsai CH (2021). A cross-sectional analysis of the association between sleep duration and osteoporosis risk in adults using, 2005–2010 NHANES. Sci Rep.

[12] Tang Y, Liu J, Feng Z, Liu Z, Wang S, Xia Y (2022). Nocturnal sleep duration and bone mineral density: a cross-sectional study of the National Health and Nutrition Examination Survey (NHANES), 2007–2014. BMC Endocr Disord.

[13] Moradi S, Shab-bidar S, Alizadeh S, Djafarian K (2017). Association between sleep duration and osteoporosis risk in middle-aged and elderly women: A systematic review and meta-analysis of observational studies. Metabolism.

[14] Ochs-Balcom HM, Hovey KM, Andrews C, Cauley JA, Hale L, Li W (2020). Short Sleep Is Associated With Low Bone Mineral Density and Osteoporosis in the Women's Health Initiative. J Bone Miner Res.

[15] Wang D, Ruan W, Peng Y, Li W (2018). Sleep duration and the risk of osteoporosis among middle-aged and elderly adults: a dose-response meta-analysis. Osteoporos Int J Establ Result Coop Eur Found Osteoporos Natl Osteoporos Found USA.

[16] Liberati A, Altman DG, Tetzlaff J, Mulrow C, Gøtzsche PC, Ioannidis JPA (2009). The PRISMA statement for reporting systematic reviews and meta-analyses of studies that evaluate healthcare interventions: explanation and elaboration. BMJ.

[17] Meneses-León J, Hernández-Salazar S, Robles-Rivera K, Tamayo-Ortiz M, Muciño-Sandoval K, Rivas-Ruiz R (2024). Association Between Changes in Sleep, Nap Duration and Bone Mineral Density in Mexican Adults. Calcif Tissue Int.

[18] Petrov ME, Liu L, Mudappathi R, Whisner CM (2024). Actigraphic sleep patterns are associated with bone turnover and bone mineral density among university students. J Sleep Res.

[19] Rassow K, Obst A, Nauck M, Völzke H, Stubbe B, Fietze I (2024). Sleep characteristics and parameters of bone turnover and strength in the adult population: results from the Study of Health in Pomerania-TREND. J Sleep Res.

[20] Chen KH, Su CM, Liu WJ, Tzeng HE, Lee CL, Tsai CH (2024). The joint effects of physical activity and sleep duration on risk of osteoporosis in Taiwanese adult population: The Taiwan Biobank Study. Osteoporos Int.

[21] Ma Q, Liu T, Li Y, Xu H, Xiao Q, Yao Q (2023). The Association Between Sleep Duration and Sleep-Related Gene Methylation with Osteoporosis in Chinese Postmenopausal Women. Rejuvenation Res.

[22] Zhao H, Zhu L, Fan L, Yang J, Hou J, Zhang G (2023). Association of nocturnal sleep duration and sleep midpoint with osteoporosis risk in rural adults: a large-scale cross-sectional study. Sleep Breath.

[23] Yamaura R, Kasahara H, Iimuro S, Yamazaki T (2023). The Association between Sleep and Bone Mineral Density: Cross-Sectional Study Using Health Check-up Data in a Local Hospital in Japan. JBMR Plus.

[24] Zeng H, Li L, Zhang B, Xu X, Li G, Chen M (2022). Relationship between sleep pattern and bone mineral density in patients with osteoporotic fracture. Ther Adv Endocrinol Metab.

[25] Cherian KE, Kapoor N, Paul TV (2022). Disrupted Sleep Architecture Is Associated With Incident Bone Loss in Indian Postmenopausal Women: A Prospective Study. J Bone Miner Res.

[26] Tang Y, Wang S, Yi Q, Xia Y, Geng B (2021). Sleep pattern and bone mineral density: a cross-sectional study of National Health and Nutrition Examination Survey (NHANES) 2017–2018. Arch Osteoporos.

[27] Pan F, Tian J, Cicuttini F, Jones G (2021). Sleep disturbance and bone mineral density, risk of falls and fracture: Results from a 10.7-year prospective cohort study. Bone.

[28] Swanson CM, Blatchford PJ, Stone KL, Cauley JA, Lane NE, Rogers-Soeder TS (2021). Sleep duration and bone health measures in older men. Osteoporos Int.

[29] Bevilacqua G, Denison HJ, Laskou F, Jameson KA, Ward KA, Cooper C (2020). Self-reported Sleep Quality and Bone Outcomes in Older Adults: Findings from the Hertfordshire Cohort Study. Calcif Tissue Int.

[30] Swanson CM, Blatchford PJ, Orwoll ES, Cauley JA, LeBlanc ES, Fink HA (2019). Association between objective sleep duration and bone mineral density in older postmenopausal women from the Study of Osteoporotic Fractures (SOF). Osteoporos Int.

[31] Marques EA, Figueiredo P, Gudnason V, Lang T, Sigurdsson G, Sigurdsson S (2017). Associations of 24-hour sleep duration and CT-derived measurements of muscle and bone: The AGES-Reykjavik Study. Exp Gerontol.

[32] Lucassen EA, De Mutsert R, Le Cessie S, Appelman-Dijkstra NM, Rosendaal FR, Van Heemst D (2017). Poor sleep quality and later sleep timing are risk factors for osteopenia and sarcopenia in middle-aged men and women: The NEO study. Miao D, ed. PLOS ONE.

[33] Saint Martin M, Labeix P, Garet M, Thomas T, Barthélémy JC, Collet P (2016). Does Subjective Sleep Affect Bone Mineral Density in Older People with Minimal Health Disorders?The PROOF Cohort. J Clin Sleep Med.

[34] Tian Y, Shen L, Wu J, Xu G, Yang S, Song L (2015). Sleep duration and timing in relation to osteoporosis in an elderly Chinese population: a cross-sectional analysis in the Dongfeng–Tongji cohort study. Osteoporos Int.

[35] Niu J, Sahni S, Liao S, Tucker KL, Dawson-Hughes B, Gao X (2015). Association between Sleep Duration, Insomnia Symptoms and Bone Mineral Density in Older Boston Puerto Rican Adults. Romigi A, ed. PLOS ONE.

[36] Cunningham TD, Di Pace BS (2015). Is Self-Reported Sleep Duration Associated with Osteoporosis?Data from a 4-Year Aggregated Analysis from the National Health and Nutrition Examination Survey. J Am Geriatr Soc.

[37] Wang K, Wu Y, Yang Y, Chen J, Zhang D, Hu Y (2015). The associations of bedtime, nocturnal, and daytime sleep duration with bone mineral density in pre- and post-menopausal women. Endocrine.

[38] Chen G, Chen L, Wen J, Yao J, Li L, Lin L (2014). Associations Between Sleep Duration, Daytime Nap Duration, and Osteoporosis Vary by Sex, Menopause, and Sleep Quality. J Clin Endocrinol Metab.

[39] Kim N, Choi HR, Kim SW, Kim BS, Won CW, Kim SY (2014). Association between Bone Mineral Density and Sleep Duration in the Korean Elderly Population. Korean J Fam Med.

[40] Kobayashi D, Takahashi O, Deshpande GA, Shimbo T, Fukui T (2012). Association between osteoporosis and sleep duration in healthy middle-aged and elderly adults: a large-scale, cross-sectional study in Japan. Sleep Breath.

[41] Fu X, Zhao X, Lu H, Jiang F, Ma X, Zhu S (2011). Association between sleep duration and bone mineral density in Chinese women. Bone.

[42] Specker BL, Binkley T, Vukovich M, Beare T (2007). Volumetric bone mineral density and bone size in sleep-deprived individuals. Osteoporos Int.

[43] Cheng CH, Chen LR, Chen KH (2022). Osteoporosis Due to Hormone Imbalance: An Overview of the Effects of Estrogen Deficiency and Glucocorticoid Overuse on Bone Turnover. Int J Mol Sci.

[44] Smit AE, Meijer OC, Winter EM (2024). The multi-faceted nature of age-associated osteoporosis. Bone Rep.

[45] Stich FM, Huwiler S, D'Hulst G, Lustenberger C (2021). The Potential Role of Sleep in Promoting a Healthy Body Composition: Underlying Mechanisms Determining Muscle, Fat, and Bone Mass and Their Association with Sleep. Neuroendocrinology.

[46] Van Cauter E, Plat L (1996). Physiology of growth hormone secretion during sleep. J Pediatr.

[47] Giustina A, Mazziotti G, Canalis E (2008). Growth Hormone, Insulin-Like Growth Factors, and the Skeleton. Endocr Rev.

[48] Juliana N, Azmi L, Effendy NM, Mohd Fahmi Teng NI, Abu IF, Abu Bakar NN (2023). Effect of Circadian Rhythm Disturbance on the Human Musculoskeletal System and the Importance of Nutritional Strategies. Nutrients.

[49] BabićLeko M, Pleić N, Gunjača I, Zemunik T (2022). Environmental Factors That Affect Parathyroid Hormone and Calcitonin Levels. Int J Mol Sci.

[50] Swanson CM (2021). Sleep Disruptions and Bone Health: What Do We Know So Far?. Curr Opin Endocrinol Diabetes Obes.

[51] MacDonald IJ, Tsai HC, Chang AC, Huang CC, Yang SF, Tang CH (2021). Melatonin Inhibits Osteoclastogenesis and Osteolytic Bone Metastasis: Implications for Osteoporosis. Int J Mol Sci.

[52] Maria S, Samsonraj RM, Munmun F, Glas J, Silvestros M, Kotlarczyk MP (2018). Biological effects of melatonin on osteoblast/osteoclast cocultures, bone, and quality of life: Implications of a role for MT2 melatonin receptors, MEK1/2, and MEK5 in melatonin-mediated osteoblastogenesis. J Pineal Res.

[53] Patel SR, Zhu X, Storfer-Isser A, Mehra R, Jenny NS, Tracy R (2009). Sleep Duration and Biomarkers of Inflammation. Sleep.

[54] Sampson HW (2002). Alcohol and Other Factors Affecting Osteoporosis Risk in Women. Alcohol Res Health.

[55] Kelly RR, Sidles SJ, LaRue AC (2020). Effects of Neurological Disorders on Bone Health. Front Psychol.

[56] Colten HR, Altevogt BM, Research I of M (US) C on SM and (2006). Extent and Health Consequences of Chronic Sleep Loss and Sleep Disorders. In: Sleep Disorders and Sleep Deprivation: An Unmet Public Health Problem.

